# Fructan Biosynthesis by Yeast Cell Factories

**DOI:** 10.4014/jmb.2207.07062

**Published:** 2022-09-08

**Authors:** Hyunjun Ko, Bong Hyun Sung, Mi-Jin Kim, Jung-Hoon Sohn, Jung-Hoon Bae

**Affiliations:** 1 Synthetic Biology & Bioengineering Research Center, Korea Research Institute of Bioscience and Biotechnology (KRIBB), Daejeon 34141, Republic of Korea; 2 Cellapy Bio Inc., Bio-Venture Center 211, Daejeon 34141, Republic of Korea

**Keywords:** Fructan, levan, inulin, fructosyltransferase, yeast, fermentation

## Abstract

Fructan is a polysaccharide composed of fructose and can be classified into several types, such as inulin, levan, and fructo-oligosaccharides, based on their linkage patterns and degree of polymerization. Owing to its structural and functional diversity, fructan has been used in various fields including prebiotics, foods and beverages, cosmetics, and pharmaceutical applications. With increasing interest in fructans, efficient and straightforward production methods have been explored. Since the 1990s, yeast cells have been employed as producers of recombinant enzymes for enzymatic conversion of fructans including fructosyltransferases derived from various microbes and plants. More recently, yeast cell factories are highlighted as efficient workhorses for fructan production by direct fermentation. In this review, recent advances and strategies for fructan biosynthesis by yeast cell factories are discussed.

## Introduction

Fructan, a natural fructose polymer, is a typical prebiotic that aids the growth of probiotics and beneficial microbes in the human intestine. Along with prebiotics, fructan is applied in various fields including foods and beverages, cosmetics, and medical and pharmaceutical industries owing to its physiochemical characteristics (water solubility, biocompatibility, biodegradability, and gel formation) and physiological effects (immunomodulation, anti-oxidant, anti-tumor, and anti-AIDS) [1, 2]. Fructans have attracted increasing attention owing to their wide application.

**Fig. 1:**
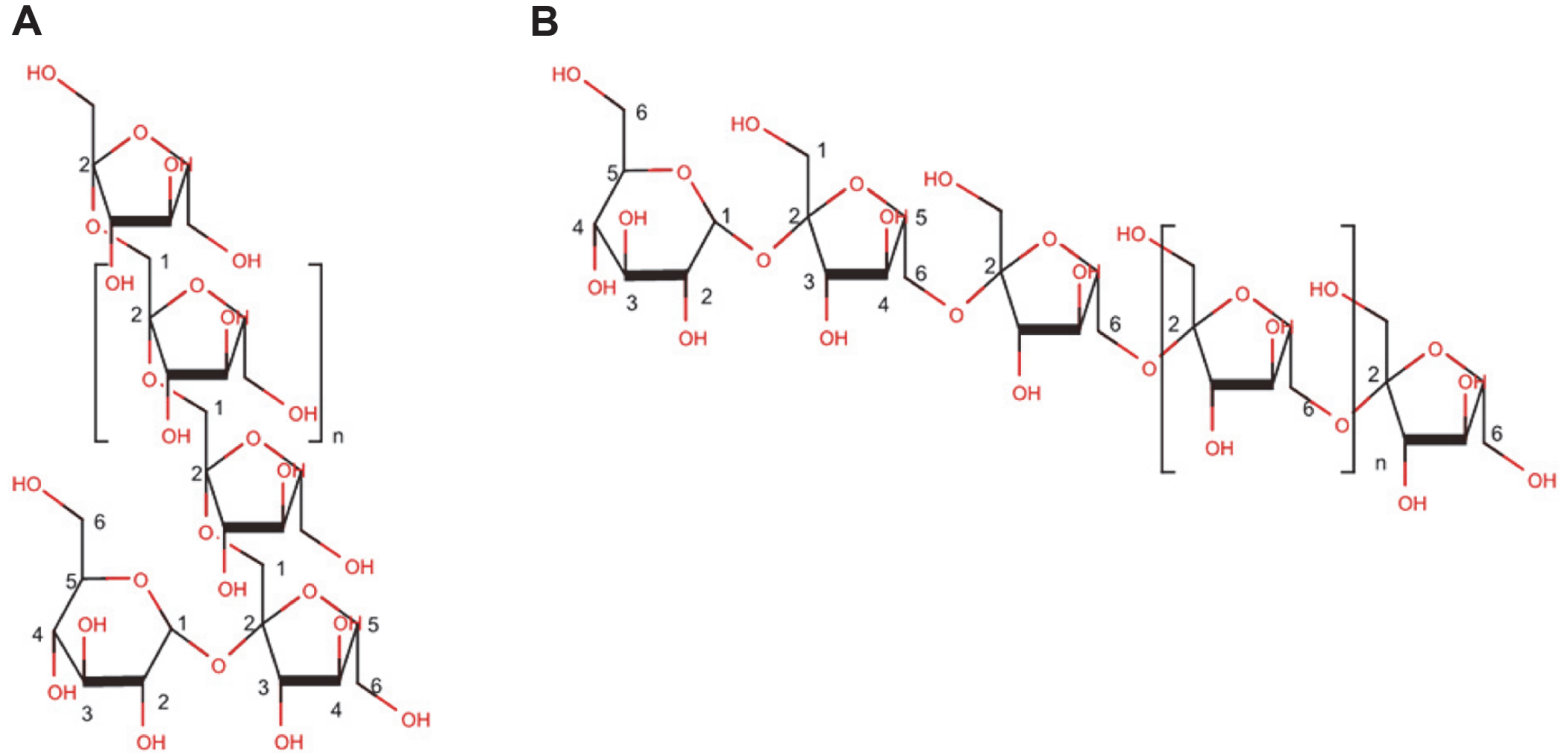
Basic structures of two fructans. Structures of inulin and levan are depicted in (**A**) and (**B**), respectively.

Fructan is classified according to two criteria namely linkage orientation between fructose units and the degree of polymerization (DP). Based on the former criterion, it was divided into two groups: inulin and levan (Fig. 1). Inulin consists of β-(2,1)-linked β-D-fructosyl units and rarely branched through β-(2,6) linkages. Levan, is β-(2,6)-linked and branched by β-(2,1) linkages. Unlike inulin, the degree of branching (DB) of levans varies up to 13% according to the means of production [3, 4]. Regarding the DP criterion, fructan is divided into three groups: short-chain fructo-oligosaccharides (scFOS, DP 2-10), medium-chain FOS (mcFOS, DP ranging to 20), and higher DP (DP > 20, inulin, and levan) [5].

The fructan was identified more than two centuries ago. In 1804, Rose isolated a substance from *Inula helenium* using hot-water extraction [6]. After that, Thomson named the substance as “Inulin” [7]. Levan, was identified in 1881 by Lippmann named as “Lävulan,” and was produced from microbial fermentation in 1901 by Greig-Smith [8]. The recent production of fructan is achieved by extraction from plant sources and biosynthesis from sucrose. The former is favored for the production of inulin-type fructan from plant tubers [9, 10]. The latter is used for the production of levan-type fructan via enzymatic conversion (fructosyltransferases; FTase, [EC 2.4.1.X]) or microbial fermentation [2].

Recently, with increased interest in healthy lifestyles, the global market for fructan has steadily increased, with an annual growth rate of > 5%, and is expected to reach approximately US$2.7 billion by 2026 [11]. Considering the increasing market size, efficient and straightforward fructan production systems have been developed. In this review, a brief overview of fructans and the recent advances in its production is provided. In summary, the natural occurrence and physiological roles of fructan and the enzymes involved in its biosynthesis are described. In the last section, we focused on the strategies of yeast cell factories to produce fructan based on earlier work.

## Fructan in Nature

Fructan is a natural carbohydrate found in various plants and microbes. In general, inulin-type fructan is found in plant systems as a reserve carbohydrate, and levan-type is abundant in microbial systems as a constituent of the exopolysaccharide (EPS) matrix. Although botanical and microbial fructans are synthesized by distinct enzyme systems, both play the same role in stress resistance.

### Botanical Fructan and the Related Enzymes

Sucrose and starch are common reserve carbohydrates in most plants, and approximately 15% of flowering plants store fructan as a reserve carbohydrate. Typically, inulin has been found in various plants, including cereals, vegetables, grasses, and decorative plants [12]. In contrast, levans are found in small quantities in only a few grass plants, mainly in their stems and leaf sheaths [13]. Chicory, dahlia, Jerusalem artichoke, and yacon are good producers of inulin because they accumulate up to 80% polysaccharides in their tubers [14, 15]. Although the quantity and quality of accumulated inulin from these crops fluctuate seasonally and regionally, chicory produces a higher DP inulin, an important quality standard for inulin than other crops [16]. Owing to its growth under harsh conditions, Jerusalem artichoke is also considered an efficient producer of inulin [17].

Fructan-producing plants grow under frost and drought and are rarely found in tropical regions [18]. The correlation between fructan and frost/drought was experimentally verified using different climate-adapted *Bromus* models and transgenic tobacco plants. *Bromus pictus* adapted to cold desert areas exhibited higher tolerance to chilling and water-deficient conditions than *Bromus auleticus* inhabiting warmer areas. The main difference between the two species is the ratio of sucrose to fructan accumulated in the roots [19]. Transgenic tobacco expressing bacterial levansucrase showed improved drought tolerance compared with wild-type plants [20]. To explain the tolerance of plants to abiotic stress induced by fructan, a membrane stabilization model was suggested. In this model, fructan molecules are bound to the polar head groups of the lipid bilayer of the membrane to block water leakage during abiotic stress such as frost and drought [21].

Botanical fructan is synthesized from sucrose by successive reactions with four types of fructosyltransferases (FTases):1-SST (sucrose:sucrose 1-fructosyltransferase, EC 2.4.1.99), 1-FFT (fructan:fructan 1-fructosyltransferase, EC 2.4.1.100), 6-SFT (sucrose:fructan 6-fructosyltransferase, EC 2.4.1.10), and 6G-FFT (fructan:fructan 6G-fructosyltransferase, EC unassigned). Edelman and Jefford proposed an inulin biosynthetic mechanism in plants that employed 1-SST and 1-FFT [22]. In this model, 1-SST transfers the fructose moiety of sucrose to another sucrose molecule, resulting in the formation of 1-kestose. Following the first enzyme reaction, 1-FFT elongates the β-(2,1) fructan chain and 6-SFT and 6G-SFT are employed to synthesize β-(2,6)-linked fructan in plants. The former conducts transfructosylation on sucrose and 6-kestose, synthesizing a higher DP levan-type fructan [23]. The latter translocates the β-(2,1)-linked fructose units of 1-kestose to the 6^th^ carbon of glucose to form neokestose [24].

### Microbial Fructan and the Related Enzymes

Microbial fructan is a constituent of the extracellular polymeric substance (EPS) matrix in biofilms. Similar to botanical inulin, microbial biofilms protect cells from various environmental stressors [25, 26]. For example, biofilms of probiotic bacteria *Bifidobacterium* and *Lactobacilli* in the intestine protect against environmental stresses, such as temperature and bacteriostatic agents [27, 28]. *Streptococcus mutans* is a well-known parasitic cavity bacterium causing dental plaques. The EPS of dental plaque contains approximately 30% fructan, protecting cells from antimicrobial materials in saliva [29].

Since the discovery of *Bacillus* sp. producing a viscous biopolymer on sucrose-containing media [8], numerous studies have been conducted on fructan produced by microorganisms, such as bacteria and fungi. In particular, levans, are widely studied in bacterial systems. To date, dozens of bacterial species are identified as levan-producers. Gram-positive bacterial levan producers such as *Bacillus* [30], *Geobacillus* [31], *Lactobacillus* [32], *Leuconostoc* [33], *Microbacterium* [34], *Paenibacillus* [35], and *Streptococcus* genus [36] and Gram-negative producers like *Erwinia* [37], *Gluconacetobacter* [38], *Halomonas* [39], *Pseudomonas* [40], *Serratia* [41], and *Zymomonas* genus [42], have been identified. The DP and DB of levans from different microorganisms vary according to production conditions such as temperature, pH, agitation speed, and substrate concentration. Gram-negative bacteria, however, produce a higher DP levan (up to several million Daltons) than gram-positive bacteria (up to several thousand Daltons) [3]. Contrary to DP, the DB of levans produced by gram-positive bacteria is higher than that produced by gram-negative bacteria [35, 43]. Some parasitic and symbiotic bacteria, such as *Streptococcus mutans* and the *Lactobacillus* genus, respectively, are reported to produce inulin-type fructan. However, unlike levans, microbial inulin is mainly produced as FOS (DP 3–5) [44, 45].

Microbial fructan synthesis is more efficient than plant systems because it is synthesized from sucrose by a single enzyme reaction. For inulin-type fructan, inulosucrase (ISRase, EC 2.4.1.9, for inulin) was used. Functional analysis of ISRase was conducted using enzymes from the *Lactobacillus* genus [45-49], but has not been applied in the industrial production of fructan due to low productivity. Therefore, the industrial production of inulin-type fructan has been performed using FTases from *Aspergillus* species, ensuring superior fructosylation activity. However, fungal FTases generally produce less than four scFOS DPs [50]. Levansucrase (LSRase, EC 2.4.1.10,) has been applied in industrial production of levan contrary to ISRase, which is a levan producing enzyme. For example, commercial levan production has been achieved using LSRases from *Bacillus subtilis* (Natural Polymers Inc., USA), *Streptococcus salivarius* (Advance Co., Ltd., Japan), and *Zymomonas mobilis* (Real Biotech Co, Ltd., Korea) [3, 51].

Interestingly, *the Lactobacillus* genus encodes for two types of FTases (ISRase and LSRase) producing inulin-and levan-type fructans, which constitute EPS. Based on this property, van Hijum *et al*. conducted structural and functional analysis of FTase from *Lactobacillus* [52]. They subdivided FTases into four domains: secretion signals, amino-terminal variable regions, catalytic domains, and carboxy-terminal variable regions. In the catalytic domain, six core sequence motifs containing a catalytic triad, two sucrose-binding sites, and an acid/base catalytic domain were suggested. In the caroboxy-terminal variable region, a well-known cell wall-anchoring motif (LPXTG) is present in both ISRase and LSRase from lactic acid bacteria and may play a role in the cell-surface display of FTases for efficient EPS production.

## Microbial Production of Fructan by Yeast Cell Factories

Yeasts are used to produce fermented beverages and foods for thousands of years [53] and have played important roles in biotechnology as model organisms, recombinant protein producers [54, 55], and cell factories for high-value biochemicals and biofuels [56, 57]. Recently, yeast has been used as a cell factory for the efficient production of fructan.

### Recombinant Expression of FTases in Yeasts

**FTase expression in *Saccharomyces cerevisiae*.** Baker’s yeast, *S. cerevisiae*, is the most widely studied in biotechnology. With a vast amount of information and well-established techniques, *S. cerevisiae* has been used as a recombinant protein producer, including various FTases.

Secretory production of microbial FTase in *S. cerevisiae* was attempted by Scotti *et al*. They expressed LSRase from *B. subtilis* (SacB) with a native signal peptide in the *S. cerevisiae* S150 strain, however, the LSRase was found in the intracellular portion as a precursor form. Interestingly, cytosolic pre-SacB showed higher levan synthetic activity than purified SacB from *B. subtilis* [58]. These authors constructed a hybrid SacB with signal peptides from yeast acid phosphatase (Pho), invertase (Suc2), and alpha-amylase from *Bacillus amyloliquefaciens*. However, recombinant SacB was found in the cytoplasm using immunofluorescence microscopy and cellular fractionation analysis [59]. In contrast to the bacterial LSRase, a fungal FTase from *Aspergillus foetidus* was successfully secreted by a native signal peptide in the *S. cerevisiae* YSH strain. The activity of the culture supernatant reached 85,200 U/L, and 1-kestose was produced from sucrose using recombinant FTase [60]. These studies imply that native bacteria and certain yeast signal peptides cannot lead recombinant LSRase to a secretory pathway in yeast.

To enhance the feasibility of secretory production of heterologous proteins in *S. cerevisiae*, Bae *et al*. developed a target protein-specific secretion signal called the translational fusion partner (TFP) screening system [61]. TFPs consist of signal peptides and propeptides facilitating the proper folding of cargo proteins in the endoplasmic reticulum (ER) and trafficking to the Golgi complex. Using a TFP screening system, Ko *et al*. developed recombinant yeasts secreting LSRases from *Rahnella aquatilis* (LsrA) and *B. subtilis* (SacB) [62, 63]. As optimal TFPs, the pre-pro regions of Uth1p and Srl1p were selected for the secretion of LsrA and SacB, respectively. By fed-batch fermentation, bioactive LsrA and SacB were produced in media with 50,000 and 3,800 U/L, respectively. Ko *et al*. reported the first secretory production of bacterial ISRase from *Lactobacillus reuteri* (*INU1*)[64]. To achieve secretory production of ISRase in yeast, truncated *INU1* variants were constructed because the carboxy-terminus of *INU1* contains a cell wall-anchoring domain. The *INU1ΔNC* variant (truncated at both the amino- and carboxy-termini) was successfully produced extracellularly using TFP. Direct fermentation of a recombinant yeast strain secreting truncated ISRase in sucrose medium produced medium-chain FOS (DP 3–20).

**FTase expression in *Pichia pastoris*.** Along with *S. cerevisiae*, the methylotrophic yeast *Pichia pastoris* has been extensively used for the production of recombinant proteins by virtue of its high cell density fermentation, high secretory capacity, and strong inducible *AOX1* promoter [65].

During the early 2000s, various FTases from many plant sources were functionally expressed in *P. pastoris*. To produce FTases acting in the β-(2,1) direction, 1-SSTs from *Festuca arundinacea*, *Lolium perenne*, and *Phleum pretense* were expressed in the X-33 strain, and conversion of 1-kestose as a major product from sucrose was verified [66–68]. 1-FFTs from *Arctium lappa*, *Triticum aestivum*, and *Viguiera discolor* were produced in the X-33 and GS115 strains, and elongation of fructosyl units on kestose produced by recombinant enzymes was confirmed [69, 70]. As to FTases acting in the β-(2,6) direction, recombinant 6-SFT from *Hordeum vulgare* showed both 6-SFT and 1-SST activity in sucrose media [71]. The bioconversion of β-(2,6) fructan from sucrose by plant FTase has been verified by Ueno *et al*. They expressed 6G-SFT from *Asparagus officinalis* using the X-33 strain and confirmed production of neokestose with a small quantity of 1-kestose from sucrose [72].

Interestingly, various microbial FTases have been extracellularly produced in *P. pastoris* by employing *S. cerevisiae* signal peptides. Trujillo *et al*. expressed LSRase from the gram-negative bacterium *Gluconacetobacter diazotrophicus* (LsdA) using the GS115 and X-33 strains. The *AOX1* promoter and PHO1 signal peptide were used for the extracellular secretion of LsdA. Through methanol-feeding fermentation, recombinant LsdA was secreted into the culture medium with a bioactivity of 740 U/L. Purified LsdA produced 1-kestose and nystose from sucrose under optimum conditions (pH 5 and 60°C) [73]. In a subsequent study, they used the *GAPDH* promoter for constitutive expression of LsdA with a mating factor alpha secretion signal in the X-33 strain and produced approximately 4,000 U/L of bioactive LSRase in the fermentation media [74]. LSRase from the gram-positive bacterium *Leuconostoc mesenteroides* (M1FT) was expressed in GS115 strain. After 120 h of batch-fed fermentation with methanol induction, bioactive M1FT (14,400 U/L) was produced in a 5-L fermenter [75]. More recently, FTases derived from *Aspergillus niger* GS610 and YZ59 were cloned and expressed in GS115 strain. Both recombinant fungal FTases were produced extracellularly by glycerol feeding and were subsequently characterized after purification [76, 77].

**FTase expression in other yeast species.** Another methylotrophic yeast, *Ogataea polymorpha* (*Hansenula polymorpha*), was used for the extracellular production of LSRase from *Z. mobilis* (*LevU*). For efficient secretory production, the signal peptide of exoinulinase from *Kluyveromyces marxianus* was fused to the amino-terminus of *LevU*, and produced 12,200 U/L of bioactive material by batch-fed fermentation after methanol induction. Recombinant *LevU* exhibited psychrophilic activity similar to that of the original enzyme [78]. As a well-known β-galactosidase producer, *Kluyveromyces lactis* has been used for the production of various industrial enzymes. Using *K. lactis* strain GG799, Spohner and Czermak succeeded in extracellular production of FTase from *Aspergillus terreus*. They introduced a codon-optimized *A. terreus* FTase gene into a pKLAC2-based vector, and recombinant FTase was secreted into the culture broth with 986,400 U/L of activity after galactose induction. The optimum conditions of the purified enzyme were pH 6 and 60°C, and FOS of DP ranging from 2 to 6 were synthesized [79]. Zhang *et al*. constructed a whole-cell catalyst to produce FOS using yeast cell-surface display of FTase from *Aspergillus oryzae*. Interestingly, they utilized the non-conventional oleaginous yeast *Yarrowia lipolytica*, used in functional food supplements and biofuel production. Cell-surface-displayed FTase showed 850 U/g of dry cell weight, and the production of FOS (DP ranging between 2 and 5) was confirmed [80].

As described above, utilization of yeast to produce recombinant proteins, including FTase, has advantages. In particular, high-yield extracellular secretions facilitate simplified downstream processes. However, most studies indicate the need for yeast-derived signal peptides for successful secretory production of FTases. In addition, there is a concern in yeast production system because glycosylation, a typical post-translational modification, may occasionally lead to undesirable changes in the bioactivity of the target protein. In practice, most recombinant FTases produced in yeast have a molecular weight, higher than the expected size, characterized by N-glycosylation (Table 1). However, to date, there are no reports on loss of fructosylation activity by glycosylated FTases produced in yeast.

### Yeast Cell Factories for Consolidated Fructan Biosynthesis

**Fig. 2:**
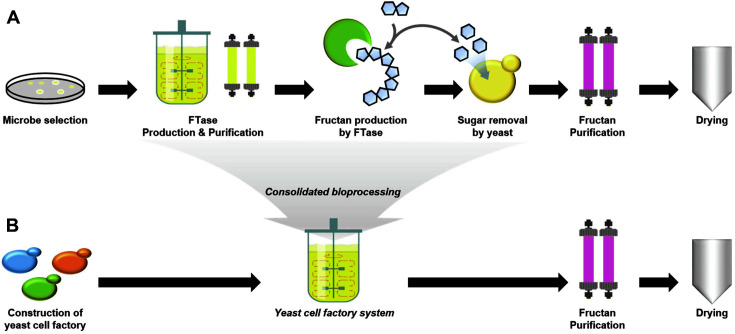
Comparison of enzymatic and yeast cell factory system for fructan production. Schematic diagrams of enzymatic and yeast cell factory systems are compared on (**A**) and (**B**), respectively.

Beyond FTase producers, yeasts have been used as cell factories for efficient production of fructan for the following reasons. First, owing to the lack of a fructan metabolism pathway, the productivity of fructan is generally high. Second, yeasts consume glucose and fructose, which are by-products released during fructan conversion from sucrose. These by-products are well-known FTase inhibitors [81, 82], and the consumption of glucose and fructose by yeast cells reduces the feedback inhibition of FTase and guarantees a high conversion yield. Furthermore, removal of monosaccharides increases the purity of the fructan. Thus, a highly efficient consolidated process can be established by simple fermentation of sucrose using recombinant yeast (Fig. 2).

One significant drawback of using yeast for fructan production is the requirement for a specific gene deletion. Most yeasts produce hydrolysis enzyme (invertase) to use sucrose as a carbon source, and the invertase produced competes with FTase, decreasing conversion yields. Therefore, the use of an invertase-deficient mutant strain is essential for increasing fructan production via the yeast system.

**Fig. 3:**
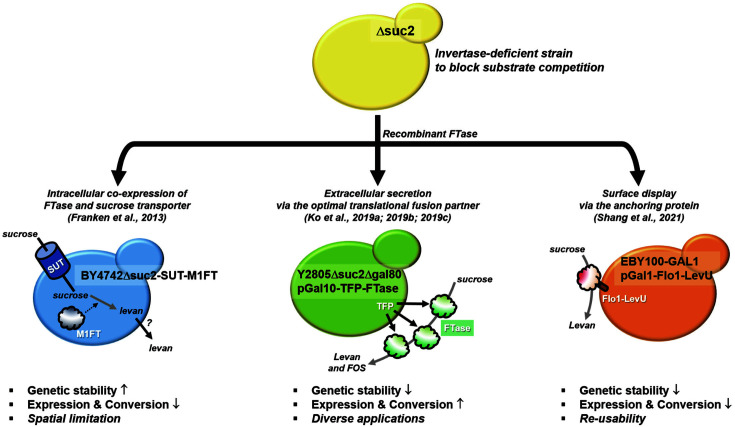
Strategies for fructan biosynthesis by yeast cell factories. Three strategies for construction of yeast cell factories for fructan biosynthesis are organized based on the reference studies. FTase, fructosyltransferase; TFP, translational fusion partner.

There are three strategies for the construction of yeast cell factories for fructan production according to the expression location of recombinant FTase: intracellular, extracellular, and cell surface display are described in Fig. 3.

The intracellular FTase expression has been demonstrated by Franken *et al*. [83]. They constructed a recombinant *S. cerevisiae* strain BY4742 intracellularly expressing a bacterial LSRase from *L. mesenteroides* (M1FT). To achieve fructan synthesis by yeast, they mimicked plant systems that accumulate fructan in their bodies using two approaches: sucrose uptake and cytosolic sucrose synthesis. The former model was constructed by co-expression of a spinach sucrose transporter (SUT), and the latter established by co-expression of a sucrose synthase from potato (SuSy). Both models showed biosynthesis of levan-type fructan in their cytosol from sucrose-containing media (YPDS, 10 g/l yeast extract, 20 g/l peptone, 30 g/l glucose, and 50 g/l sucrose). Between the two models, the SUT-expressing yeast cell factory showed higher levan (7.75 g/l titer and 15.5% conversion yield). In this system, heterologous genes are integrated into the host genome to guarantee high genetic stability. However, the cytosolic accumulation has a spatial limitation of the cell and requires an extra cell disruption process, leading to an unsatisfactory production titer for the scale-up process.

The yeast cell factory accomplishing extracellular FTase expression can overcome the major hurdle of the intracellular fructan production because it is synthesized in culture media by secreted FTase. However, this system requires FTase to be secreted efficiently. Ko *et al*. employed the TFP technique to achieve hypersecretion of recombinant FTase. For the levan producer, *S. cerevisiae* Y2805Δgal80Δsuc2 strains expressing LSRase from *R. aquatilis* were constructed, and 76 g/l levan was converted from 191 g/l sucrose-containing media (39.8%conversion yield) with undetectable amounts of monosaccharides in a 50-L fermenter. The productivity was 3.17 g/l/h, being the most efficient system ever reported at the fermenter scale. Owing to the high-speed agitation, the molecular weight of the produced levan decreased to 810,000 Da compared to enzymatic conversion (over 3,750,000 Da) [62, 84]. In addition, high titer (102.9 g/l) production of levan (molecular weight approximately 140,000 Da) was demonstrated at the flask level by employing recombinant yeast secreting LSRase from *B. subtilis* [63]. Inulin-type FOS was also demonstrated by Ko *et al*. as a production for other fructan-types. They developed a yeast cell factory that secretes bacterial ISRase from *L. reuteri* using the TFP technique. Inulin production was performed in a 5-L fermenter by culturing recombinant yeast cells in sucrose-containing media. The highest FOS titer was 152.6 g/l with 400 g/l sucrose, and the highest conversion efficiency was 42.7% with 300 g/l sucrose (128 g/l FOS). By ion chromatography analysis, the produced FOS was identified as mcFOS ranging from DP to 2–20; suggesting one-step production of mcFOS instead of a two-step process, involving extraction of inulin from plants and subsequent enzymatic treatment [64].

Whole-cell biocatalysis is an attractive system for the following reasons. First, this system can solve the spatial limitations of intracellular production. Second, the whole-cell catalyst system ensures high stability against abiotic stresses, such as high temperature and metal ion inhibition. Third, owing to the immobilization effect, the cell factory can be used repeatedly. Fructan synthesis via yeast cell surface-displaying FTase was reported by Shang *et al*. They constructed *S. cerevisiae* EBY100-GAL1 strain that displayed levansucrase from *Z. mobilis* (*LevU*) [85]. The highest titer of levan produced was 34 g/l, with a molecular weight of 670,000 Da. In addition, the yeast cell factory demonstrated six cycles of reusability, suggesting high stability and economical production. However, they used an invertase-expressing yeast strain that simultaneously utilized sucrose by FTase and invertase. The authors also discussed the importance of invertase use during levan production to increase conversion yield.

## Conclusion and Future Perspective

Demand and interest for fructan have increased over the time, owing to its versatile applications. However, the high production cost of fructan is a major hurdle in its application. Currently, the enzymatic conversion system is favored in the industrial production of fructan because the system is well-established, easy to scale-up, and above all, free of the safety issue of the living modified organism (LMO). Nevertheless, a cell factory system (not limited to yeast) has been consistently proposed and developed owing to the overwhelming productivity required for economic production. Approximately 70 microbial species, including various yeast species, have been authorized as LMO. Therefore, LM yeasts proven to be safe for humans and the environment will be the best option for the efficient production of fructan.

## Abbreviations

DP, degree of polymerization; DB, degree of branching; FOS, fructo-oligosaccharides; scFOS; short-chain FOS; mcFOS, medium-chain FOS; EPS, exopolysaccharides; FTase, fructosyltransferases; 1-SST, sucrose:sucrose 1-fructosyltransferase; 1-FFT, fructan:fructan 1-fructosyltransferase; 6-SFT, sucrose:fructan 6-fructosyltransferase; 6G-FFT, fructan:fructan 6G-fructosyltransferase; ISRase, inulosucrase; LSRase, levansucrase.

## Figures and Tables

**Table 1:** Recombinant FTases produced in yeast species.

Expression host		Target FTase		Genetic tools		Expression			References

Species	Strain	Type	Origin	Promoter	Signal peptide	Location	Size (kDa)	Yield (U/l)	
*Saccharomyces cerevisiae*	S150	LSRase	*Bacillus subtils*	ADH1	Native	Intra-cellular	53^a^	720	[58]
	IWA	LSRase	*Bacillus subtils*	ADH1	PHO5	Intra-cellular	53^b^	460	[59]
	YSH	FTase	*Aspergillus foetidus*	ADH1	Native	Extra-cellular	60^c^	85,200	[60]
	BY4742	LSRase	*Leuconostoc mesenteroides*	PGK	w/o	Intra-cellular	n/d	n/d	[83]
	2805	LSRase	*Rahnella aquatilis*	GAL10	UTH1	Extra-cellular	47^a^	50,000	[62]
	2805	LSRase	*B. subtils*	GAL10	SRL1	Extra-cellular	51^c^	3,800	[63]
	2805	ISRase	*Lactobacillus reuteri*	GAL10	UTH1	Extra-cellular	60^c^	220,000	[64]
*Pichia pastoris*	GS115	LSRase	*Gluconacetobacter diazotrophicus*	AOX1	PHO1	Extra-cellular	60^a^	740	[73]
	X-33	LSRase	*G. diazotrophicus*	GAP	MFα	Extra-cellular	60^a^	4,000	[74]
	GS115	LSRase	*L. mesenteroides*	AOX1	MFα	Extra-cellular	66^a^	14,400	[75]
	GS115	FTase	*A. niger*	AOX1	MFα	Extra-cellular	97-120^a^	2,294,700	[76]
	GS115	FTase	*A. niger*	AOX1	MFα	Extra-cellular	n/d	1,020,000	[77]
*Kluyveromyces lactis*	GG799	FTase	*A. terreus*	LAC4	MFα/	Extra-cellular	80^a^	986,400	[79]
*Hansenula polymorpha*	A16	LSRase	*Z. mobilis*	MOX	INU	Extra-cellular	50^c^	12,200	[78]
*Yarrowia lipolytica*	CGMCC7326	FTase	*A. oryzae*	HP4D	PIR1	Cell-surface	n/d	850 U/g of DCW	[80]

w/o, without; n/d, not described

^a^Protein size with glycosylation

^b^Protein size of unprocessed precursor form

^c^Protein size without glycosylation

**Table 2:** Recombinant yeast cell factories for fructan biosynthesis.

Yeast cell factories			Production condition					Product					References

FTase/Location	Key genotype	Strategy	Scale (L)	Sucrose (g/l)	pH	T^a^ (°C)	Agitation (rpm)	Type	MW^b^ (Da)	DP^c^	Titer (g/l)	Yield^d^ (%)	
LmM1FT/Intracellular	BY4742Δsuc2-SUT	Sucrose uptake	0.1	50	6.5	30	n/d	Levan	n/a	280	7.8	15.5	[83]
RaLsrA/Secretion	Y2805Δgal80Δsuc2	Optimal secretion	30	191	5.5	30	300	Levan	810,000	n/a	76.0	39.8	[62]
BsSacB/Secretion	Y2805Δgal80Δsuc2	Optimal secretion	0.05	250	5.5	30	180	Levan	140,000	n/a	102.9	41.2	[63]
ZmLevU/Surface	EBY100-GAL1	Re-usability	0.02	400	6.0	35	shaking	Levan	690,000	n/a	34.0	8.5	[85]
LrInu/Secretion	Y2805Δgal80Δsuc2	Optimal secretion	2	400	5.5	30	900	FOS	n/a	2-20	152.6	38.2	[64]

n/a, not applicable

^a^T, temperature

^b^MW, molecular weight

^c^DP, degree of polymerization

^d^Yield was calculated as the amount of fructan produced relative to the initial substrate (w/w)
